# Abundance, diversity, and composition of root-associated microbial communities varied with tall fescue cultivars under water deficit

**DOI:** 10.3389/fmicb.2022.1078836

**Published:** 2023-01-12

**Authors:** Jialin Hu, Grady Miller, Wei Shi

**Affiliations:** Department of Crop and Soil Sciences, North Carolina State University, Raleigh, NC, United States

**Keywords:** drought, cultivar, Actinobacteria, Basidiomycota, turfgrass, plant-microbe interactions

## Abstract

The plant breeding program has developed many cultivars of tall fescue (*Festuca arundinacea*) with low maintenance and stress tolerance. While the root-associated microbial community helps confer stress tolerance in the host plant, it is still largely unknown how the microbiota varies with plant cultivars under water stress. The study aimed to characterize drought-responsive bacteria and fungi in the roots and rhizosphere of different tall fescue cultivars. Intact grass-soil cores were collected from six cultivars grown in a field trial under no-irrigation for 3 years. Tall fescue under irrigation was also sampled from an adjacent area as the contrast. Bacterial and fungal communities in roots, rhizosphere, and bulk soil were examined for abundance, diversity, and composition using quantitative-PCR and high-throughput amplicon sequencing of 16S rRNA gene and ITS regions, respectively. Differences in microbial community composition and structure between non-irrigated and irrigated samples were statistically significant in all three microhabitats. No-irrigation enriched Actinobacteria in all three microhabitats, but mainly enriched Basidiomycota in the root endosphere and only Glomeromycota in bulk soil. Tall fescue cultivars slightly yet significantly modified endophytic microbial communities. Cultivars showing better adaptability to drought encompassed more relatively abundant Actinobacteria, Basidiomycota, or Glomeromycota in roots and the rhizosphere. PICRUSt2-based predictions revealed that the relative abundance of functional genes in roots related to phytohormones, antioxidant enzymes, and nutrient acquisition was enhanced under no-irrigation. Significant associations between *Streptomyces* and putative drought-ameliorating genes underscore possible mechanics for microbes to confer tall fescue with water stress tolerance. This work sheds important insight into the potential use of endophytic microbes for screening drought-adaptive genotypes and cultivars.

## 1. Introduction

Drought has been established as the greatest culprit limiting agricultural production because it can stunt the growth of crops by adversely impacting crop physiology and reducing soil nutrient availability (Bista et al., 2018). Decades of studies support that soil- and phytomicrobiomes are able to help ameliorate plant drought resistance *via* regulating phytohormone production, scavenging reactive oxygen species (ROS), and improving plant nutrient acquisition (Farooq et al., 2009; Naylor and Coleman-Derr, 2017; Xi et al., 2018; Inbaraj, 2021). However, the extent and manner by which microbes confer plant drought resistance are plant species- and microhabitat-specific (Naylor et al., 2017; Wang et al., 2020).

Microbial community composition and diversity vary substantially among root endosphere, rhizosphere, and the adjacent bulk soil (Schlaeppi et al., 2014; Naylor et al., 2017; Dastogeer et al., 2020). Such a niche differentiation often comes with different microbial roles in sustaining plant growth under water-stressed environment. Root endophytic microbes are likely more related to the regulation of phytohormones and antioxidants (Xu et al., 2017), but mycorrhizal fungi are heavily involved in transporting water and nutrients to their host plants (Parniske, 2008). While drought can decrease the overall activity of soil microbes for mining nutrients, recurrent water stress may shape the soil microbiome with an enhanced capability of nutrient (e.g., nitrogen) acquisition (Lau and Lennon, 2012; Canarini et al., 2021). Niche differentiation in the rhizosphere and root endosphere microbiomes have been shown to vary with plant species and genotypes (Berg and Smalla, 2009; Bouffaud et al., 2014; McPherson et al., 2018; Dastogeer et al., 2020). Empirical experiments on economic crops, such as barley, maize and potato also revealed cultivar-level effects of a particular plant species (Peiffer et al., 2013; Bulgarelli et al., 2015; Fitzpatrick et al., 2018). Though contributing relatively small changes (2–8% of the variance) in microbial communities (Peiffer et al., 2013; Bulgarelli et al., 2015; Edwards et al., 2015; Wang et al., 2020), cultivar-level effects may have profound implications in the plant breeding program, microbial biotechnology development and strategic management planning. For example, Gram-positive, oligotrophic bacteria (e.g., Actinobacteria, Chloroflexi, Firmicutes) are often found to be enriched under drought (Chodak et al., 2015; Naylor and Coleman-Derr, 2017; Breitkreuz et al., 2021; Sun et al., 2022). If their abundance is proven to be associated with drought tolerance behavior of cultivars, these bacteria may be used as the phenotype to help reliably screen stress-tolerance cultivars.

Turfgrass occupies ~2% of the continental U.S. land and provides important roles in environmental protection and many benefits to humans as well (Milesi et al., 2005). It is among the largest irrigated crops in the United States (Mayer et al., 1999) and often needs intensive management. Compared to other cool-season grasses, tall fescue with deep and extensive root systems, can better adapt to and survive short drought periods. Still, a number of cultivars and breeding lines of low maintenance and stress tolerance have been released over time. In the present study, we aimed to evaluate tall fescue cultivars for drought adaptive ability from the perspective of belowground microbial communities, considering that plant-associated microbiota is the ‘extended phenotype’ of the host (van Opstal and Bordenstein, 2015; Dawkins, 2016). Our objectives were to (1) investigate cultivar-based changes in the root endosphere, rhizosphere and bulk soil microbial communities, and (2) construe the potential function of belowground microbiota in sustaining healthy tall fescue under water-stressed environment. We also attempted to propose elite microbes in helping confer tall fescue drought resistance by linking microbial taxa with the cultivar quality rating under drought. This work is urgently needed because knowledge development of host plant microbial ecology would not be comprehensive in the absence of turfgrass, a major anthropogenic ecosystem.

## 2. Materials and methods

### 2.1. The study site and turfgrass/soil sample collection

Tall fescue (*Festuca arundinacea*) is generally deemed as a low maintenance grass, being more tolerant to drought, heat, and disease than other cool-season grasses; yet new cultivars have been continuously released to improve this kind of trait. A low-maintenance experiment was set up in October 2018, at the Lake Wheeler Turfgrass Field Laboratory of NC State University to evaluate how 28 cultivars of tall fescue would perform under no irrigation, i.e., precipitation as the sole source of water for plant growth. Because of the shortage of water supply, meaning that precipitation was largely less than evapotranspiration, no irrigation led to water deficit in plants, with grass canopy being brown in hot summers. Tall fescue cultivars were randomly arranged into a total of 112 1.37 m × 1.37 m plots by a complete block design with four replications/blocks. The soil at this site was classified as Cecil sandy loam (fine, kaolinitic, thermic Typic Kanhapludults) with a 9% slope. All plots were professionally managed for fertilization and herbicide applications in the same manner. In late September 2021, we took samples from six tall fescue cultivars (DaVinci, Fayette, Maestro, Matisse, Michelangelo, and Rockwell) of first three blocks, leading to a total of 18 intact grass-soil cores (5 cm diameter × 10 cm height, one core per plot × six cultivars × three replicates). Four intact grass-soil cores were also collected as controls from the adjacent irrigated area of a multi-cultivar blend of tall fescue (Rain Dance, Coronado, and Cumberland), where overhead irrigation was run every other day at approximately 0.2 to 0.25 inches of water per irrigation. This rate of irrigation was able to meet the water requirement of tall fescue growing in the area. As a result, grass canopy remained dark green in hot summers. Together, 22 intact grass-soil cores were transported to the laboratory in an ice-packed cooler and then stored at a 4°C refrigerator.

### 2.2. Processing of root, rhizosphere, and bulk soil samples

The grass-soil cores were smashed using a sterilized trowel to separate grass roots from bulk soils. The bulk soil was sieved through 2-mm mesh. Roots and the rhizosphere soil were separated using a modified bleach-washing protocol (McPherson et al., 2018; Xia et al., 2021). For each sample, the grass roots with tightly attached soil were carefully excised using a sterilized scissor and placed into a labeled 50 ml falcon tube containing 35 ml autoclaved phosphate buffer (6.33 g/l NaH_2_PO_4_, 8.5 g/l Na_2_HPO_4_, pH = 6.5) and surfactant (200 μl/l, Silwet L-77). The roots were picked out using a sterilized tweezer after shaking for 2 min. Then, the roots were re-cleaned one more time as described above, blotted dry on a paper towel, and placed into a new 50 ml tube. The rhizosphere soil was collected by centrifuging the slurries at 3000 × *g* for 5 min at room temperature and discarding the supernatant. The roots were then sequentially washed with 35 ml 50% bleach (0.01% Tween 20), 70% ethanol, and sterilized water by shaking for 1 min. After that, the roots were washed with sterilized water for three times, blotted dry on a paper towel, and stored in a new 50 ml tube. The grass roots, rhizosphere soil, and ~ 2 g bulk soil samples were stored at −20°C for DNA extraction. The remainder of the bulk soils were stored at 4°C for soil physicochemical properties analyses.

### 2.3. Soil physicochemical properties measurement

Soil gravimetric water content was determined by measuring the difference between the weight of fresh soil and oven-dried (105°C, 48 h) soil and calculating the water lost as a percentage of the dried soil weight. Soil pH was measured in 1:2.5 soil (g)/water (mL) suspension using pH electrode (Fisher Scientific, Pittsburgh, PA, United States). Soil NH_4_^+^- and NO_3_^−^-N were quantified by a microplate reader (BioTek Instruments Inc., Winooski, VT, United States) using Berthelot reaction-based (Rhine et al., 1998) and Vanadium (III) chloride-based (Doane and Horwáth, 2003) spectrophotometric methods, respectively, after extracting 5 g fresh soil in 20 ml of 1 M KCl solution and filtering through a Whatman filter paper #1.

### 2.4. DNA extraction and quantitative PCR for microbial abundance

Microbial metagenomic DNA of bulk soil (0.5 g), rhizosphere soil (0.1–0.4 g), and grass roots (0.1–0.3 g) were extracted using the FastDNA Spin Kit for Soil (MP Bio, Solon, OH, United States). The quantity and quality of DNA were measured using the NanoDrop spectrophotometer (NanoDrop Technologies, Wilmington, DE, United States). Extracted DNA samples were stored at −20°C for downstream analyses.

The total bacterial and fungal abundances were measured using Femto Bacterial and Fungal DNA Quantification kits (Zymo Research Corp., CA, United States) with primer pairs 8F/357R for bacteria (8F: 5’-AGAGTTTGATCCTGGCTCAG-3′ and 357R: 5’-CTGCTGCCTCCCGTAGG-3′) and ITS-1F/ITS-2R for fungi (ITS-1F: 5’-CTTGGTCATTTAGAGGAAGTAA-3′ and ITS-2R: 5′- GCTGCGTTCTTCATCGATGC-3′), respectively, through quantitative PCR (qPCR) method on a CFX96 Optical Real-Time Detection System (Bio-Rad, Laboratories Inc., Hercules, CA, United States). The averaged qPCR amplification efficiencies for bacteria and fungi were around 88 and 90%, respectively.

### 2.5. DNA library preparation and amplicon sequencing

Microbial metagenomic DNA samples were diluted to 5 ng μL^−1^ for the amplification of bacterial 16S rRNA genes and fungal ITS regions. PCR amplifications were performed with Illumina-compatible adapter-added primer pairs targeting bacterial V3-V4 region (341F: 5’-CCTACGGGNGGCWGCAG-3′ and 805R: 5’-GACTACHVGGGTATCTAATCC3’) and fungal ITS1-ITS2 (F_KYO2: 5′- TAGAGGAAGTAAAAGTCGTAA-3′ and R_KYO2: 5’-TTYRCTRCGTTCTTCATC-3′), respectively, in a 25-μL PCR mixture containing 12.5 μl KAPA HiFi HotStart ReadyMix (KAPA Biosystems, Wilmington, MA, United States), 2.5 μl template DNA, and 5 μl of each primer (1 μM). The thermal cycling conditions were initial denaturation at 95°C for 3 min; 30 cycles of 95°C for 30 s, 55°C for 30 s, and 72°C for 30 s for bacteria and 98°C for 30 s, 51°C for 15 s, and 72°C for 30 s for fungi; followed by final elongation at 72°C for 5 min. Then, the PCR products were purified with AMPure XP beads (Beckman Coulter Genomics, Danvers, MA, United States), and added unique index sequences/barcodes (Nextera XT Index Kit, Illumina, San Diego, CA, United States) in a 50-μL reaction containing 25 μl KAPA HiFi HotStart ReadyMix, 5 μl of each forward/reverse index primer, 5 μl purified PCR product, and 10 μl PCR grade nuclease-free water. After the second clean-up with AMPure XP beads, the final indexed PCR products were quantified by a NanoDrop spectrophotometer, diluted to 40 nM, pooled together, mixed thoroughly, and sequenced on Illumina Miseq platform (300 × 2 PE) (Illumina, San Diego, CA, United States). The raw sequencing data were deposited to NCBI Sequence Read Archive (SRA) Database, BioProject ID: PRJNA884421.

### 2.6. Bioinformatics and statistical analyses

The primers were removed from raw sequencing data by Cutadapt (v 3.5) (Martin, 2011). Then, the sequences were processed in R (v 4.1.2) (R Core Team, 2021) with DADA2 (v 1.22.0) pipeline (Callahan et al., 2016) for dereplication, error model training, forward and reverse reads merging, chimeras removal, and generation of the amplicon sequence variants (ASVs) table. After that, the ASV table and their unique sequences file were imported into QIIME2 (v 2021.11) (Bolyen et al., 2019) for diversity and taxonomy analysis after singletons removal. The Greengenes database (v 13.8) (DeSantis et al., 2006) and UNITE database (v 8.3) (Nilsson et al., 2019) were used for the annotation of bacterial and fungal ASVs, respectively. The alpha diversity indices (observed ASVs, Chao1 index, Pielou’s evenness, and Shannon index) and beta diversity were analyzed at the sequence depth of 17,552 reads for bacteria and 32,958 reads for fungi. Weighted-UniFrac and Bray–Curtis dissimilarity matrices were used for bacterial and fungal beta diversity analysis, respectively. The putative copy numbers of KEGG ortholog genes were predicted based on the bacterial ASVs at the sequencing depth of 17,552 using PICRUSt2 (Douglas et al., 2020). When discussing microbial responses to water deficit at the species level, operational taxonomic units (OTUs), defined as 97% nucleotide sequence similarity, were used as the proxies of species in bacterial and fungal communities (Konstantinidis and Tiedje, 2005; Green et al., 2017).

The Shapiro–Wilk test was used to check the data normality. For normally distributed data, one-way ANOVA with LSD *post hoc* test was used to test the effects of microhabitats, cultivar., and irrigation on soil physicochemical properties, microbial abundances, and alpha diversity indices. For non-normally distributed data, the difference between irrigation and no-irrigation was tested by Mann–Whitney U test, and the difference among cultivars was tested by Kruskal–Wallis test with Dunn’s *post hoc* test. Differences in beta diversity among different groups were assessed by permutational multivariate analysis of variance (PERMANOVA) and visualized by principal coordinates analysis (PCoA). Spearman’s rank correlation analysis was performed to assess the relationships among different parameters, with coefficients|ρ| > 0.7 0.5 ≤ |ρ| < 0.7, 0.3 ≤ |ρ| < 0.5|ρ| < 0.3 representing good, moderate, weak, and negligible associations, respectively.

## 3. Results

### 3.1. Soil physicochemical properties and microbial abundances

Soil physicochemical properties, except NH_4_^+^-N, were significantly affected by irrigation treatment, but not by cultivars of tall fescue (*p* < 0.05, Table 1). Compared to irrigation, no-irrigation led to a ~ 4.1- and a ~ 2.6-fold decrease in soil moisture and NO_3_^−^-N, respectively, and a ~ 1.1-fold increase in soil pH. Under no-irrigation, soil NH_4_^+^- and NO_3_^−^-N averaged at 8.74 and 16.13 μg g^−1^ soil, respectively, with relatively large variations among individual soil samples (43.8% the coefficient of variation for NH_4_^+^-N and 57.2% for NO_3_^−^-N).

**Table 1:** Soil properties as affected by irrigation treatments and tall fescue cultivars under no irrigation.

Group	Moisture (%)	pH	Ammonium	Nitrate	(μg N g^−1^ soil)	
**Irrigation treatment**				
Irrigation	41.54 ± 2.03a	6.14 ± 0.01b	12.63 ± 4.36	41.67 ± 10.65a
No-irrigation	10.22 ± 0.58b	6.66 ± 0.02a	8.74 ± 0.90	16.13 ± 2.17b
**Cultivars**				
DaVinci	9.79 ± 0.58	6.67 ± 0.03	8.09 ± 0.93	12.14 ± 1.95
Fayette	11.78 ± 1.41	6.69 ± 0.01	8.81 ± 0.42	17.90 ± 3.09
Maestro	9.91 ± 0.88	6.63 ± 0.09	9.37 ± 4.46	20.81 ± 11.60
Matisse	12.66 ± 2.62	6.67 ± 0.04	7.77 ± 1.37	15.11 ± 4.44
Michelangelo	8.61 ± 0.26	6.71 ± 0.05	10.36 ± 3.98	15.51 ± 6.71
Rockwell	8.54 ± 0.61	6.61 ± 0.01	8.03 ± 0.65	15.32 ± 2.62

The significant difference between irrigation treatments or among cultivars was tested by Kruskal–Wallis one-way ANOVA with Dunn-Bonferroni *post hoc* tests (α = 0.05) and marked as different letters.

**Figure 1:**
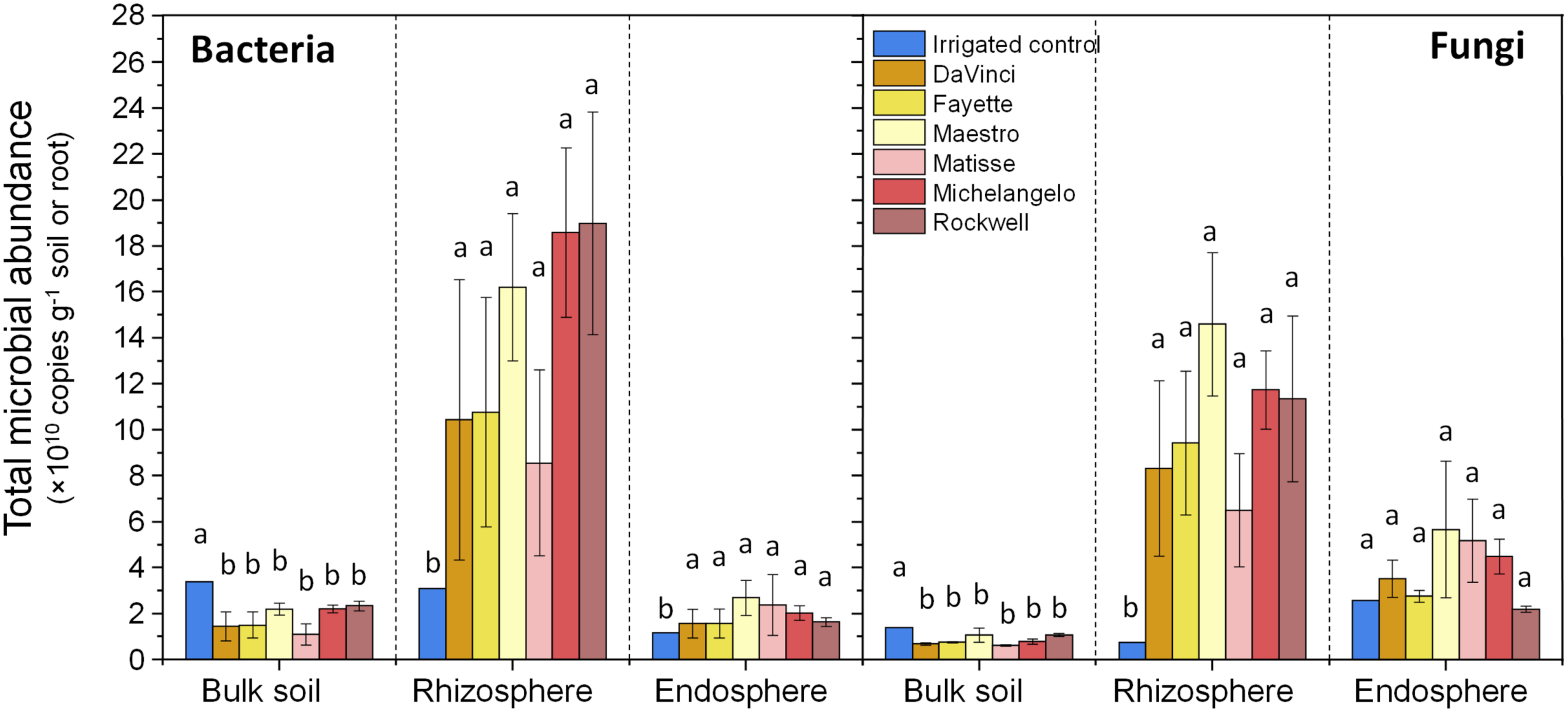
Total bacterial (16S rRNA gene copy numbers) and fungal (ITS copy numbers) abundance in the bulk soil, rhizosphere, and root endosphere of tall fescue samples under different irrigation and cultivars. Abundances are expressed as means ± standard errors. Different letters indicate significant differences at *p* < 0 05.

The abundance of bacteria and fungi both significantly varied among microhabitats (*p* < 0.001, Figure 1). Bacterial abundance was higher in the rhizosphere and comparably lower in the root endosphere and bulk soil, while fungal abundance in the rhizosphere and root endosphere was similar but greater than that in bulk soil. Regardless of microhabitats, both bacterial and fungal abundances were statistically insignificant among the six cultivars. Both bacteria and fungi were more abundant under irrigation than no-irrigation in bulk soil (*p* < 0.01 for both), but the reverse was true for the rhizosphere (*p* < 0.05 for bacteria, *p* < 0.01 for fungi) and root endosphere (*p* < 0.05 for bacteria), except for endophytic fungi. In addition, bacterial and fungal abundances in the rhizosphere were significantly and negatively correlated with soil moisture, while those in bulk soil were markedly correlated with soil pH and inorganic N (Supplementary Figure 1). Bacterial abundances in the rhizosphere and root endosphere were strongly and positively correlated, but fungal abundances in the three microhabitats were unrelated. Within each microhabitat, bacterial and fungal abundances showed strong and positive correlations (Supplementary Figure 1).

### 3.2. Microbial diversity and community composition

Alpha diversity metrics significantly differed among microhabitats, being greatest in bulk soil or rhizosphere but lowest in the root endosphere for both bacterial and fungal communities (Table 2). Cultivars slightly yet significantly affected the alpha diversity metrics for bacteria only in bulk soil, with DaVinci and Rockwell having the greatest richness (i.e., observed ASVs and Chao1) but lowest evenness. Nonetheless, Shannon diversity index varied from ~8.5, the lowest in Matisse to ~8.9, the greatest in Maestro. Compared to irrigation, no-irrigation significantly increased the richness of bacterial communities in rhizosphere and root endosphere and also Shannon diversity index of the bacterial community in rhizosphere. However, no-irrigation decreased the evenness of bacterial communities in bulk soil and rhizosphere as well as Shannon diversity index of the fugal community in bulk soil (Table 2).

**Table 2:** Alpha-diversity metrics of bacterial and fungal communities as affected by microhabitats (bulk soil, rhizosphere, and root endosphere), irrigation treatments, and different cultivars of tall fescue.

	ASVs	Chao1	Evenness	Shannon
**Microhabitats** ^**#**^				
Bulk soil_B_	854 ± 31a	899 ± 35a	0.904 ± 0.002a	8.776 ± 0.048a
Rhizosphere_B_	507 ± 25b	526 ± 27b	0.905 ± 0.002a	8.105 ± 0.066b
Endosphere_B_	303 ± 11c	314 ± 12c	0.814 ± 0.005b	6.695 ± 0.065c
Bulk soil_F_	558 ± 11a	564 ± 12b	0.713 ± 0.008a	6.501 ± 0.083a
Rhizosphere_F_	649 ± 24a	672 ± 24a	0.725 ± 0.009a	6.764 ± 0.111a
Endosphere_F_	298 ± 10b	308 ± 10c	0.665 ± 0.007b	5.462 ± 0.077b
**Irrigation treatments** ^**&**^				
Irrigation_BL-B_	804 ± 45	822 ± 48	0.915 ± 0.001a	8.827 ± 0.072
No-irrigation_BL-B_	866 ± 36	917 ± 41	0.901 ± 0.002b	8.764 ± 0.058
Irrigation_RH-B_	364 ± 29b	367 ± 30b	0.916 ± 0.001a	7.784 ± 0.106b
No-irrigation_RH-B_	534 ± 23a	556 ± 25a	0.903 ± 0.003b	8.165 ± 0.067a
Irrigation_EN-B_	251 ± 12b	254 ± 12b	0.835 ± 0.008	6.652 ± 0.085
No-irrigation_EN-B_	311 ± 11a	324 ± 12a	0.811 ± 0.005	6.702 ± 0.075
Irrigation_BL-F_	593 ± 35	600 ± 34	0.751 ± 0.016	6.915 ± 0.196a
No-irrigation_BL-F_	552 ± 11	559 ± 12	0.707 ± 0.008	6.432 ± 0.083b
**Cultivars** ^¶^				
DaVinci	994 ± 55a	1,062 ± 71a	0.892 ± 0.005b	8.876 ± 0.048ab
Fayette	795 ± 55b	853 ± 66ab	0.897 ± 0.001ab	8.635 ± 0.086b
Maestro	907 ± 35ab	959 ± 35ab	0.909 ± 0.002a	8.928 ± 0.037a
Matisse	711 ± 14b	740 ± 157b	0.909 ± 0.005a	8.545 ± 0.276c
Michelangelo	791 ± 46b	822 ± 43b	0.904 ± 0.001ab	8.700 ± 0.072b
Rockwell	972 ± 9a	1,032 ± 11a	0.895 ± 0.002b	8.881 ± 0.029ab

Different letters indicate significant differences at *p* < 0.05 within respective comparison categories. Only results with significant differences were reported.

# Subscripts B and F represent bacteria and fungi, respectively. Microhabitat effects were compared within the bacterial and fungal kingdom, respectively.

& Subscripts BL, RH, and EN denote bulk soil, rhizosphere, and endosphere, respectively. Irrigation effects were compared within individual microhabitats.

¶ Cultivar effects were compared for bacteria in bulk soil only.

Microhabitat, irrigation, and their combination significantly influenced bacterial and fungal communities, with microhabitat being the primarily factor (PERMANOVA *R^2^* = 0.664 and *p* = 0.001 for bacteria; *R^2^* = 0.282 and *p* = 0.001 for fungi) and irrigation being the secondary factor (*R^2^* = 0.096 and *p* = 0.001 for bacteria; *R^2^* = 0.088 and *p* = 0.001 for fungi) (Supplementary Table 1). Separate analysis for no-irrigation samples also showed that cultivar was a key driver for structuring fungal (*R*^2^ = 0.128 and *p* = 0.001) and bacterial (*R^2^* = 0.036 and *p* = 0.058) communities. It should be noted that variations in the bacterial community were relatively large within replicates, leading to the statistical test *p* value was slightly greater than the often-used cutoff value *p* = 0.050 for significance. Nonetheless, we considered the test result was marginally significant.

**Figure 2:**
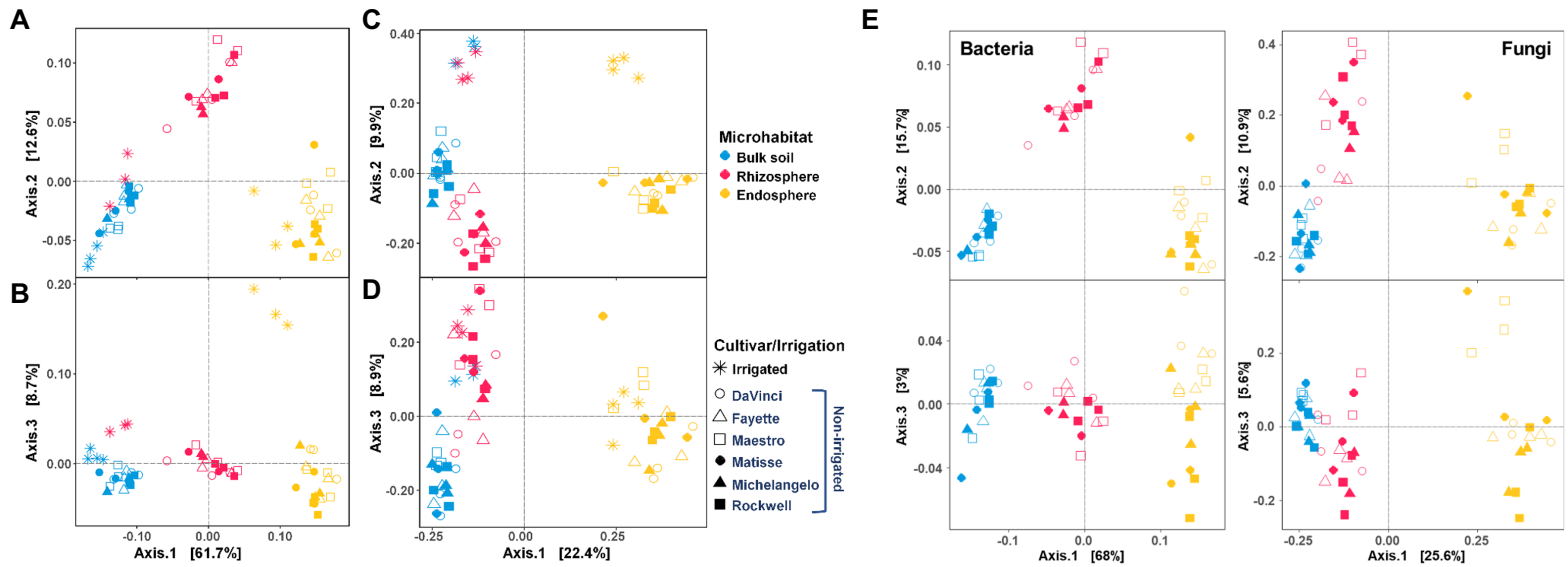
Principal coordinates analysis (PCoA) of bacterial **(A,B)** and fungal **(C,D)** communities in the bulk soil (blue), rhizosphere (pink), and root endosphere (yellow). The inset **(E)** is the PCoA of bacterial and fungal communities under no-irrigation only. Irrigation and cultivar treatments are indicated by symbols.

Principal coordinates analysis (PCoA) revealed clear separations of bacterial communities between three microhabitats (the root endosphere, rhizosphere and bulk soil) (Figures 2A,B), while fungal communities were mainly distinct between the root endosphere and rhizosphere (or bulk soil) (Figures 2C,D). Both bacterial and fungal communities under no-irrigation were well-separated from those under irrigation (Figure 2), but bacterial communities showed less response to irrigation treatments in bulk soil than in the rhizosphere and root endosphere. Furthermore, bacterial communities in the rhizosphere and bulk soil were closer under irrigation, but far distance under no-irrigation (Figures 2A,B). Under no-irrigation, bacterial and fungal communities were both separated by cultivars mainly in the endosphere (PCoA axis 3 of Figure 2E). The bacterial communities in DaVinci, Fayette, and Maestro were clustered together but were distinct from those in the other three cultivars; the fungal communities in Maestro were different from other cultivars.

### 3.3. Preferential microbial taxa under water deficit and in different cultivars

**Figure 3:**
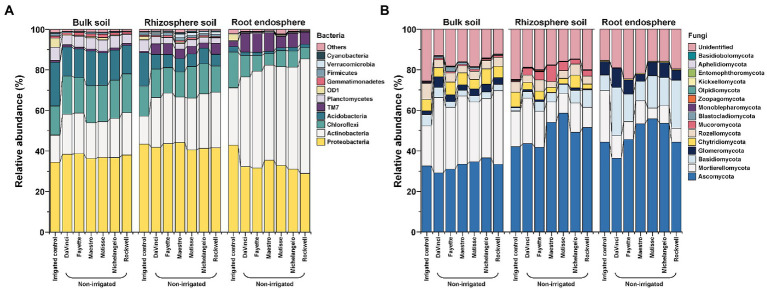
Relative abundance of the bacteria **(A)** and fungi **(B)** at the phylum level as affected by microhabitats, irrigations, and cultivars.

Irrespective of microhabitats, irrigation treatments and cultivars, the bacterial community was dominated by the phyla Proteobacteria and Actinobacteria (Figure 3A). Proteobacteria in the rhizosphere and bulk soil were affected little by irrigation treatments, averaging at ~43% in the rhizosphere and ~ 36% in bulk soil, but significantly reduced in the root endosphere by no-irrigation, i.e., ~ 32% compared to ~43% under irrigation. Actinobacteria were enriched by no-irrigation in all three microhabitats and peaked at ~49% in the root endosphere. In detail, no-irrigation led to ~1.8-, 1.8-, and 1.5-fold increase in relative abundance of Actinobacteria in the root endosphere, rhizosphere, and bulk soil, respectively, compared to irrigation. TM7 was another enhanced phylum by no-irrigation in the rhizosphere and root endosphere. Enrichment in the relative abundance by no-irrigation was also manifested in most sub-level taxa of Actinobacteria and TM7 in the root endosphere, and rhizosphere, but less in bulk soil (Supplementary Figure 2). Further, enrichment by no-irrigation in the relative abundance of Actinobacteria, TM7 and their major sub-level taxa, including Actinobacteria (class), TM7-3 (class), Actinomycetales (order), Streptomycetaceae (family), and *Streptomyces* (genus) in the root endosphere varied significantly with cultivars (Figure 3A; Supplementary Figure 2A). However, variations in Actinobacteria and TM7 between cultivars were reciprocal, with Actinobacteria being more abundant in Rockwell, Michelangelo, and Matisse, but TM7 being more enriched in DaVinci, Fayette, and Maestro.

The fungal community was dominated by the phylum Ascomycota in all three microhabitats, but the second most abundant phylum shifted from Mortierellomycota in bulk soil and the rhizosphere to Basidiomycota in the root endosphere (Figure 3B). No-irrigation significantly enhanced the relative abundance of the phylum Basidiomycota as well as some sub-level taxa of the phylum Ascomycota (e.g., Eurotiomycetes (class), Chaetothyriales (order), Herpotrichiellaceae (family), and *Exophiala* (genus)) in the root endosphere and rhizosphere (*p* < 0.05) (Supplementary Figures 3A,B). However, the relative abundance of phyla Mortierellomycota and arbuscular mycorrhizal fungi (AMF) Glomeromycota as well as their sub-level taxa were elevated in no-irrigation in bulk soil (*p* < 0.05) (Supplementary Figure 3C). Cultivar effects were also significant; for example, Davinci and Rockwell were more pronounced in promoting the relative abundance of Basidiomycota in the root endosphere and of Mortierellomycota and Basidiomycota in the rhizosphere soil as well (Figure 3B). Davinci was also better in promoting AMF Glomeromycota and its dominant sub-level taxa in bulk soil (Figure 3B; Supplementary Figure 3C).

**Figure 4:**
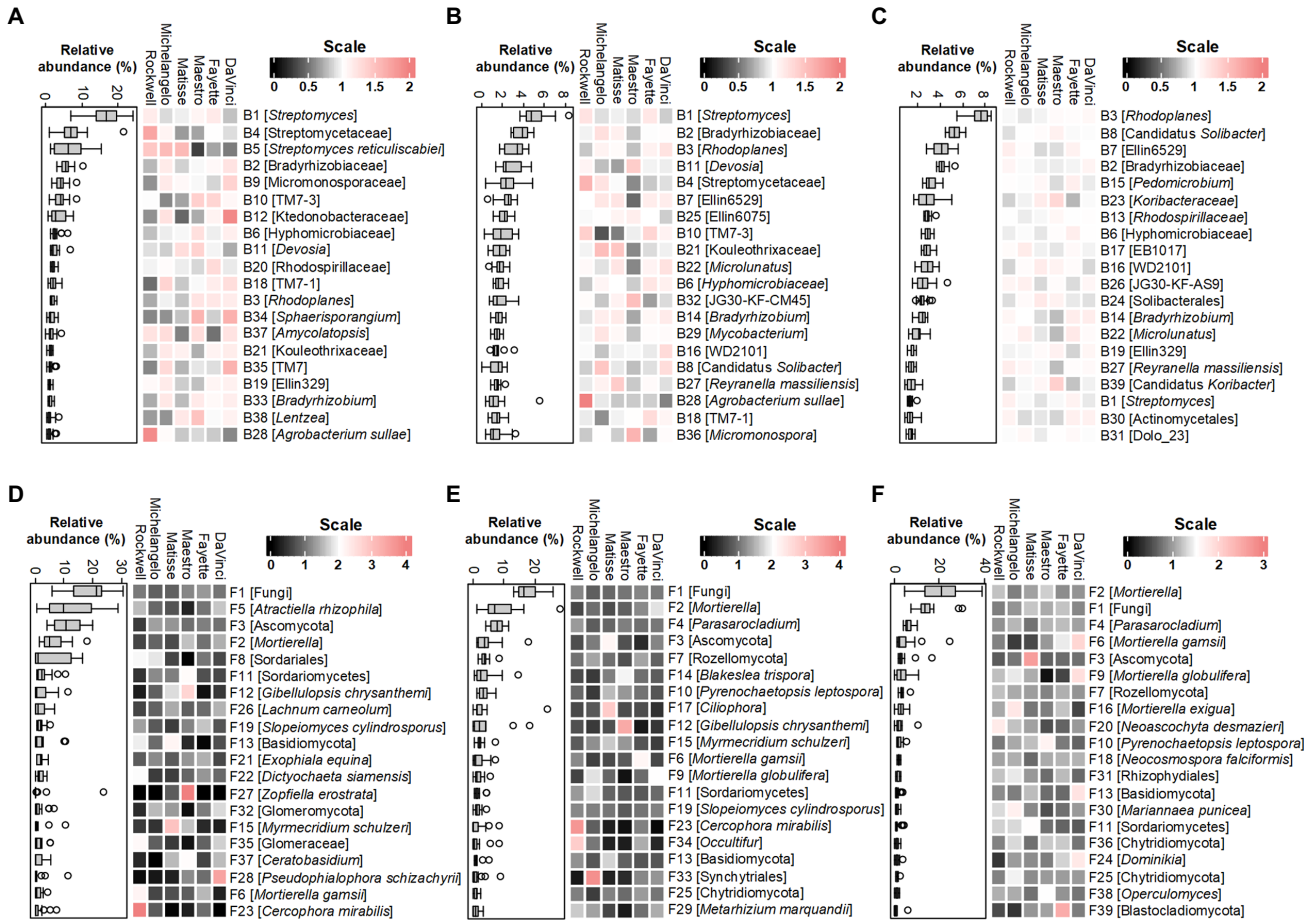
Heatmaps of normalized abundances of top 20 bacterial OTUs and fungal OTUs, respectively, in the root endosphere **(A,D)**, rhizosphere **(B,E)** and bulk soil **(C,F)** under no-irrigation. Normalization was made by dividing the relative abundance by the average value across six cultivars. Box plots represent the average values of relative abundances. The lowest-level taxonomic name of each OTU is shown in the square brackets. Complete taxonomic names are given in Supplementary Table 2.

The major bacterial and fungal species (equivalent to OTUs) under no-irrigation distributed unevenly among the six cultivars within each microhabitat (Figure 4). The top 20 abundant bacterial OTUs in the root endosphere, rhizosphere, and bulk soil occupied about 70, 43, and 57% of the total bacterial communities, respectively. The corresponding percentage for fungal OTUs were ~ 79%, ~ 73%, and ~ 79% (see Supplementary Table 2 for detailed OTUs abbreviations and taxonomic information). In general, bacterial OTUs belonging to the phylum Actinobacteria (7 of top 20 abundant OTUs) were more selective in the root endosphere, accounting for ~39% of the total bacterial abundance as compared to ~12% in the rhizosphere and ~ 8% in bulk soil, at the expense of reverse distribution in the relative abundance of the phylum Proteobacteria, which was more selective in bulk soil (~ 26%) but less in the rhizosphere (~ 16%) and root endosphere (~ 18%). Specifically, all the top 3 bacterial OTUs in the root endosphere (B1, B4, and B5) belonged to the Actinobacterial family, Streptomycetaceae, and were relatively more abundant in the cultivar Rockwell and less in DaVinci. The B5, *Streptomyces reticuliscabiei* was also less represented in Fayette and Maestro (Figure 4A). Similarly, Actinobacteria (5 of top 20 abundant OTUs) in the rhizosphere varied with cultivars, with B1 of *Streptomyces* being more abundant in Rockwell and Fayette than other cultivars and B4 of Streptomycetaceae most abundant in Rockwell, followed by Matisse and Michelangelo (Figure 4B). By contrast, the top 20 abundant bacterial OTUs in bulk soil generally showed less variations among cultivars and only 4 of them belonged to Actinobacteria (Figure 4C).

Unlike bacterial OTUs, the top 20 fungal OTUs varied largely among replicates of individual cultivars in all three microhabitats, thereby reducing the statistical power of testing variations among different cultivars (Figures 4D–F). Still, we observed some apparent changes for members of Basidiomycota, Mortierellomycota, and Glomeromycota. In the root endosphere, for example, F5 (*Atractiella rhizophila*) was more abundant in Rockwell and DaVinci and F13 (Basidiomycota) was less abundant in Fayette and Maestro than in others (Figure 4D). The relative abundance of F12 (*Gibellulopsis chrysanthemi*) was most abundant in Maestro in both root endosphere and rhizosphere (*p* < 0.05) (Figures 4D,E). F34 (Occultifur) was most abundant in Rockwell in the rhizosphere, whereas F2 and F9 (Mortierellomycota) were most abundant in DaVinci (Figure 4E). F24 (*Dominikia*), affiliating to the AMF Glomeromycota was most abundant in DaVinci and least abundant in Rockwell in bulk soil (Figure 4F). However, cultivar variations at the OTUs level were less consistent with those at the phylum level.

### 3.4. Putative functional genes

**Figure 5:**
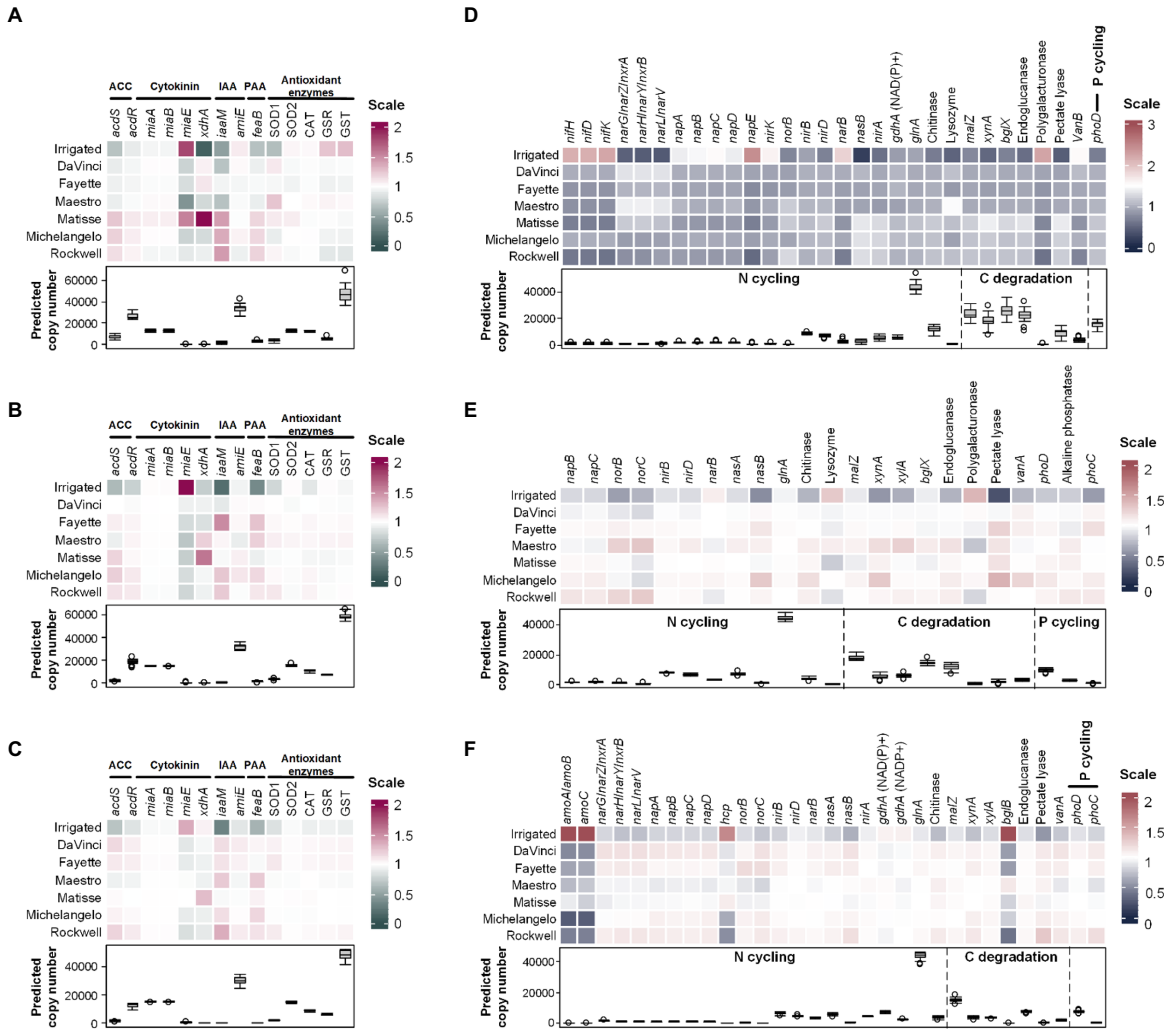
Heatmaps of normalized copy numbers of putative bacterial functional genes involved in production or regulation of phytohormones, antioxidants, and nutrient cycling at a sequence depth of 17,552. **(A–C)** are the phytohormone and antioxidants related genes in the root endosphere, rhizosphere, and bulk soil, respectively. **(D–F)** represent the nutrient cycling-related genes in the root endosphere, rhizosphere, and bulk soil, respectively. Only the genes that had significant differences between irrigation and no-irrigation were shown. Box plots show the average copy numbers of putative genes across all samples. ACC, 1-aminocyclopropane-1-carboxylic acid deaminase; IAA, indole-3-acetic acid; PAA, phenylacetic acid.

A total of 64 bacterial functional genes were examined for variations with irrigation treatments and different cultivars, 14 of which were involved in the regulation of phytohormones and antioxidants, and the others were related to nitrogen transformations, carbon degradation, and phosphate solubilization. In general, the relative abundances of these functional genes varied most in the root endosphere (Figure 5). No-irrigation significantly (*p* < 0.05) enhanced genes encoding 1-aminocyclopropane-1-carboxylic acid (ACC) deaminase (*acdS*), auxin analog indole-3-acetic acid (IAA) biosynthesis (*iaaM*), phenylacetate (PAA) biosynthesis (*feaB*), and antioxidant enzyme Cu/Zn superoxide dismutase (SOD1). No-irrigation also promoted the abundance of xanthine dehydrogenase-encoding gene *xdhA* that is involved in the oxidative breakdown of the metabolites of cytokinin but decreased the GSR (glutathione disulfide reductase) and GST (glutathione S-transferases) genes that are related to cellular antioxidant defense in the root endosphere (Figure 5A). In addition, it promoted the abundance of catalase-encoding gene CAT in the rhizosphere and bulk soil (*p* < 0.05) (Figures 5B,C). In contrast, the relative abundance of *miaE*, the gene encoding for a hydroxylase to catalyze synthesis of hydrophobic methylthiolated cytokinins, was stimulated by irrigation in all three microhabitats (*p* < 0.05) (Figures 5A–C). In the root endosphere, *acdS*, *acdR*, *iaaM*, and *feaB* were more abundant in cultivars Matisse, Michelangelo, and Rockwell than the others (Figure 5A). Matisse ranked top in the phytohormone-related genes, except *amiE*; Maestro was more advantageous in an antioxidant enzyme-encoding gene SOD1 (Figure 5A). In the rhizosphere soil, Maestro ranked top in the antioxidant enzyme-encoding genes (Figure 5B). DaVinci, Fayette, and Rockwell were more advantageous in both ACC deaminase encoding genes (acdS and acdR) and antioxidant enzyme-encoding genes than the others in bulk soil (Figure 5C). No-irrigation stimulated most of carbon-, nitrogen- and phosphorus-regulating genes in the rhizosphere and bulk soil (Figures 5E,F) but a few in the root endosphere (Figure 5D), including *nirD*, *nasB*, and *nirA* for dissimilatory and assimilatory nitrate reduction to ammonium, *glnA* for ammonium assimilation, chitinase-encoding gene for extracellular nitrogen mineralization, *malZ*, *bglX*, *xynA* and pectate lyase-encoding gene for respective degradation of starch, cellulose, hemicellulose and pectin, and *phoD* for phosphate solubilization. Abundances of these genes in the root endosphere were grater in cultivars Matisse, Michelangelo, and Rockwell than the others (Figure 5D). Cultivars also showed differences, with DaVinci generally being less stimulated but Maestro being more stimulated than others in the rhizosphere (Figure 5E). The nutrient cycling-related genes showed less variations among cultivars in bulk soil, but still were less abundant in Maestro and Matisse than the others as a whole (Figure 5F).

The relative abundances of endophytic bacterial OTUs and drought-responsive functional genes were well associated. For example, OTU B5 (classified as *Streptomyces reticuliscabiei*) was positively correlated with the ACC deaminase gene *acdS* (ρ = 0.909, *p* < 0.01) and *acdR* (ρ = 0.787, *p* < 0.01) and the biosynthesis gene of IAA (*iaaM*) (ρ = 0.950, *p* < 0.01) and PAA (*feaB*) (ρ = 0.849, *p* < 0.01). OTUs B34 of *Sphaerisporangium* and B38 of *Lentzea* were positively correlated with the superoxide dismutase gene SOD1 (ρ = 0.624 and 0.686, respectively, *p* < 0.01). OTU B5 also positively linked to nitrogen-cycling genes (e.g., *nirD*, *nasB*, *nirA*, *glnA*), chitinase-encoding gene, carbon degradation-related genes (e.g., *malZ*, *xynA*, *bglX*), endoglucanase- and pectate lyase-encoding genes, and alkaline phosphatase-encoding gene (*phoD*) (ρ > 0.600, *p* < 0.01). In addition, positive relationships were found between the dissimilatory NO_3_^−^ reduction genes (*nar*) and OTU B9 of family Micromonosporaceae (ρ > 0.500, *p* < 0.01) and OTU B34 of *Sphaerisporangium* (ρ > 0.700, *p* < 0.01).

## 4. Discussion

Tall fescue, a cool-season perennial C_3_ bunch-type turfgrass, grows vigorously in spring and fall but may go dormant in hot and dry summer days or wintertime when temperatures drop below 50°F. Indeed, the quality of all the six tall fescue cultivars under no-irrigation had gone down moderately in late winter/early spring and severely in the middle of August (Supplementary Figure 4). We were not surprised by grass turning brown under no-irrigation because of the low precipitation but high temperature from the end of July to the middle of August. Measured in early October, soil moisture under no-irrigation was only ~10%, three-fold lower than the soil moisture under irrigation, suggesting that grasses suffered water stress. Obviously, cultivars (or genotypes) differed in resilience from the summer dormancy, with Rockwell performing best and Fayette and Maestro performing worst. The visual quality ratings of tall fescue cultivars were fairly-well related to changes in microbial community compositions in the root endosphere and/or rhizosphere (Supplementary Table 3). The best performed cultivar harbored relatively more abundant Actinobacteria, Basidiomycota, or Glomeromycota in roots, compared to poorly performed cultivars.

Our data further highlights the significant role of plant host genotype in structuring the root-associated microbial community, despite being generally weak, relative to microhabitats (i.e., plant compartments) and environmental stressors (e.g., water stress) (Naylor et al., 2017; Wang et al., 2020; Huang et al., 2022). We demonstrated that both bacteria and fungi were clustered mainly by microhabitats. Although fungal communities are generally considered to be drought resistant (de Vries et al., 2018; Yang et al., 2020), water stress was the critical factor to alter bacterial as well as fungal communities, particularly in the root endosphere and rhizosphere. This phenomenon was also documented in other studies (Hartmann et al., 2017; Naylor et al., 2017; Naylor and Coleman-Derr, 2017; Xie et al., 2021). Just the same, the modulating effects of genotype/cultivar on the microbial community have been documented for several crops, e.g., barley, maize, and rice (Peiffer et al., 2013; Bulgarelli et al., 2015; Xu et al., 2019). Our work augments turfgrass to the list, showing that both bacterial and fungal communities, particularly those in the root endosphere and rhizosphere were affected by cultivars under water stress. This is likely due to different stress metabolisms and thus root exudate profiles among genotypes/cultivars (Shi et al., 2011; Badri et al., 2013).

Water stress appears to have less impact on microbial alpha diversity than on microbial community structure (Bachar et al., 2010; Armstrong et al., 2016; Tóth et al., 2017). It tends to change community structure by elevating the relative abundance of drought-tolerant microbes rather than outright abolishing drought-intolerant ones (Naylor and Coleman-Derr, 2017). This perhaps explained the lack of change in richness but a slight decrease in evenness of the bulk soil bacterial community under no-irrigation. In contrast, no-irrigation increased richness of root-associated bacterial communities, likely because of enhanced capability by root exudates in recruiting microbes from soil. Root exudates are often chemically diverse, such as soluble sugars, amino acids and organic acids and thus may help recruit diverse functional bacteria from soil for plant adaptation to drought (Shi et al., 2011). Greater species richness generally improve the metabolic capacity of microbiota and benefits the host plant to resist environmental stresses and prevent diseases (Nautiyal and Dion, 2008). Of the six cultivars examined, DaVinci and Rockwell harbored most diverse root-associated bacteria and showed greatest resiliency from the summary dormancy under no-irrigation. Our data suggest that root-associated bacterial species richness could be used as a metric for screening drought adaptive genotypes/cultivars of tall fescue.

Microbial community composition was significantly impacted by water stress and varied among cultivars. We showed that Actinobacteria were not only more abundant under no-irrigation than irrigation but also greatest in the root endosphere of the best-performed cultivar under water stress, Rockwell. Such a compositional shift suggested that Actinobacteria were the key bacteria for coping tall fescue with water stress. In fact, the relative abundances of endophytic Actinobacteria and its dominant members, e.g., *Streptomyces*, were positively correlated with the putative abundance of bacterial functional genes, encoding enzymes that may catalyze phytohormone production (e.g., ACC deaminase and auxins). By sequestering and cleaving plant-produced ACC, the immediate precursor of phytohormone ethylene, ACC deaminase can help reduce the level of ethylene in the plant and therefore promote plant drought tolerance (El-Tarabily, 2008). Root endophytic bacteria might also help promote plant drought adaptation through nutrient acquisition, given that endophytic Actinobacterial taxa, such as the abundant B5 (*Streptomyces reticuliscabiei*), B9 (Micromonosporaceae) and B34 (*Sphaerisporangium*) were positively associated with putative functional genes involved in nitrogen transformations (e.g., nitrate reduction to ammonium, ammonium assimilation, and extracellular nitrogen mineralization) and phosphate solubilization, The capability of *Streptomyces* to alleviate drought on plants has been verified *via* several *in-vivo* studies; mechanic pathways, including the production of ACC deaminase, siderophores, and IAA, the scavenging of ROS, or the solubilization of minerals have been rudimentarily established by *in-vitro* trait screening of isolates and/or transcriptional monitoring (El-Tarabily, 2008; Yandigeri et al., 2012; Anwar et al., 2016; Garcia et al., 2018; Wang et al., 2018; Xu et al., 2018; Abbasi et al., 2020). Other members of Actinobacteria have also been found to positively respond to water stress, despite few *in-vitro* and *in-vivo* tests on plant-growth-promoting traits under drought. For example, the relative abundance of Micromonosporaceae increased by up to 300% with a sequential reduction of precipitation in German forest ecosystems (Felsmann et al., 2015). Our observation that drought enriched endophytic Actinobacteria, particularly *Streptomyces* of tall fescue has also been documented in many other plants (Yandigeri et al., 2012; Naylor et al., 2017; Naylor and Coleman-Derr, 2017; Xu and Coleman-Derr, 2019; Munoz-Ucros et al., 2021; Wipf et al., 2021; Xie et al., 2021; Hu et al., unpublished manuscript^1^). This widespread phenomenon implies that species of the phylum Actinobacteria are likely elite bacteria for improving plant drought tolerance and may be used as the phenotypes of plant genotypes for quick and reliable screening of drought-tolerant cultivars in the plant breeding program.

If under drought, tall fescue-growth-promoting endophytic microbes solely belonged to Actinobacteria, DaVinci would be expected among the worst cultivars, because its roots contained the lowest abundance (i.e., 16S rRNA gene copy numbers × relative abundance) of Actinobacteria. However, DaVinci was found to be healthier than Fayette and Maestro under drought, suggesting that other microbes also contributed. DaVinci differed from Fayette and Maestro mainly in the mycorrhizal and endophytic fungal community, with DaVinci more enriched in Basidiomycota and Glomeromycota. Actually, DaVinci was comparable to Rockwell, the best drought-tolerant cultivar., in terms of the relative abundance of Basidiomycota and Glomeromycota. Thus, we considered that Basidiomycota and Glomeromycota were the elite fungi for improving tall fescue drought tolerance. Taking advantage of penetrating pores inaccessible to roots and redistributing water from moist to dry areas, Glomeromycota help alleviate drought on plants by improving the supply of water and nutrients to their host plants (Parniske, 2008; Ignacio Querejeta et al., 2012; Poudel et al., 2021). Glomeromycota can also influence plant physiological and biochemical adjustments to water stress (Cheng et al., 2021). By comparing the physiological and biochemical traits of tall fescue with and without the biofertilizer Glomeromycota, for example, Ehsan Mahdavi et al. (2018) demonstrated that under water stress, Glomeromycota-inoculated tall fescue carried more phosphorus, osmolytes, and antioxidant enzymes (Ehsan Mahdavi et al., 2018). Such an improvement is often considered as the sign of plants for better coping with abiotic stressors (Anjum et al., 2011). Another line of evidence that we were led to believe the importance of Glomeromycota in conferring tall fescue with drought tolerance was the lowest relative abundance of Glomeromycota in Maestro well-matched with its poor performance under drought.

Root-associated Basidiomycota might also play critical roles in coping tall fescue with water stress because the relative abundance of Basidiomycota was the second to Ascomycota and was consistently increased by no-irrigation, although the magnitude was cultivars-dependent. Dominance of Basidiomycota in roots has also been reported in other herbaceous plants, such as rice and creeping bentgrass (Le Van et al., 2017; Andreo-Jimenez et al., 2019). Our results were in concert with those of Lagueux et al. (2020), showing that Basidiomycota accounted for ~20% of root endophytic fungi on average across several grasslands in the United States. Authors also reported that the most drought-responsive OTU belonged to Basidiomycota and was increased by 200%. Further, the fungal compositional change, particularly the enrichment of members of Basidiomycota helped explain grass drought tolerance (Lagueux et al., 2020). Basidiomycota likely confer the tolerance of tall fescue against drought *via* osmolyte regulation, ROS scavenging, or water retention in the host (Verma et al., 2022). We noticed that root-associated Basidiomycota responded to water stress more pronouncedly at higher taxonomic ranks, e.g., the phylum level and less at the OTU-level. Still, variations in the relative abundance at both phylum and OTU levels among cultivars fairly-well paralleled with the quality ratings of cultivars under water stress. The lower the relative abundance of Basidiomycota and its OTU F13 a cultivar had, the less healthy a cultivar was under drought. Here, we need to highlight that Basidiomycota were unlikely the conserved endophytes that confer drought tolerant to all turfgrass species. Our previous work on bermudagrass turf showed obvious response of Basidiomycota to water stress in the rhizosphere and bulk soil but less response in the root endosphere (Hu et al., unpublished manuscript).^1^ However, it should be noted that the species and function of these putative elite microbes related to turfgrass drought resistance need to be confirmed through further laboratory (e.g., isolation and pot experiment) and field experiments (e.g., field inoculation) (Tao et al., 2020; Zhou et al., 2022).

Besides impacts on microbial community compositions, water firmly influenced community-level properties. Both bacterial and fungal population densities, measured as 16S rRNA and ITS gene copy number, respectively, reduced with water stress in bulk soil but surged in the rhizosphere and/or root endosphere. This was likely because water stress shifted photosynthates more towards roots to enlarge the reservoir of osmotically active compounds for osmotic adjustment (Hasibeder et al., 2015). Moderate drought might also increase the proportion of carbon allocation to the rhizosphere (Karlowsky et al., 2018; Williams and de Vries, 2020). The significant association of rhizospheric bacterial and fungal population densities with soil moisture further implied microbial dependence on drought-stressed root physiological activities. In bulk soil, drought was expected to limit diffusion and thus the supply of carbon and nutrients to microbes. However, soil bacterial and fungal population densities were mainly correlated with soil pH and inorganic nitrogen rather than soil moisture. Perhaps, drought had cascading effects over other soil processes that decoupled its direct linkage with soil microbes.

## 5. Conclusion

This work uses different cultivars of tall fescue to address a basic question in the plant breeding program: whether the root-associated microbes can be viewed as the phenotypes of plant genotype for helping screen stress-tolerant crop cultivars. A few bacterial and fungal taxa in the roots and the rhizosphere soil, including Actinobacteria, Basidiomycota, and Glomeromycota were found to positively respond to water stress; yet magnitude varied among cultivars, suggesting their suitability for appraising cultivar-specific stress fitness. This inference was also supported by concerted change between the relative abundance of drought-responsive microbial taxa and the visual quality score of tall fescue cultivars. The tight connection between drought-responsive *Streptomyces* and putative genes encoding for phytohormone regulation and ROS scavenging underscores the possible mechanics of microbes for conferring tall fescue with water stress tolerance. Differences in drought-responsive bacterial and fungal taxa were also discussed. Drought-responses are quite obvious from the phylum to genus level for Actinobacteria, but mainly at the phylum level for mycorrhizal and endophytic fungi. In addition, drought-responses of Actinobacteria appear widespread, while drought responses of Basidiomycota may not be observed in all turfgrasses. More work is needed for understanding how ecological factors interfere with the development of the root-associated Basidiomycota and Glomeromycota under drought.

## Data availability statement

The datasets presented in this study can be found in online repositories. The names of the repository/repositories and accession number(s) can be found at: https://www.ncbi.nlm.nih.gov/genbank/, PRJNA884421.

## Author contributions

JH: contributed to data collection. JH and WS: contributed to data analysis and manuscript preparation. All authors were involved in research idea development, experimental setup, and manuscript revisions. All authors contributed to the article and approved the submitted version.

## Funding

This research was funded by North Carolina Turfgrass Center for Research and Education.

## Conflict of interest

The authors declare that the research was conducted in the absence of any commercial or financial relationships that could be construed as a potential conflict of interest.

## Publisher’s note

All claims expressed in this article are solely those of the authors and do not necessarily represent those of their affiliated organizations, or those of the publisher, the editors and the reviewers. Any product that may be evaluated in this article, or claim that may be made by its manufacturer, is not guaranteed or endorsed by the publisher.

## Footnotes

^1^Hu, J., Cyle, K. T., Miller, G., and Shi, W. (unpublished). Water deficits shape the microbiome of bermudagrass roots to be Actinobacteria rich [in review].
