# Supplementary material for: Abundance, diversity, and composition of root-associated microbial communities varied with tall fescue cultivars under water deficit
Source: Front Microbiol. 2023 Jan 12;13:1078836. doi: 10.3389/fmicb.2022.1078836 (PMC9878326; doi:10.3389/fmicb.2022.1078836)
Supplement: Supplementary file 1 [file Data_Sheet_1.PDF]

Supplementary Material

1 Supplementary Tables and Figures

1.1 Supplementary Tables

Supplementary Table 1. Results of PERMANOVA for the effects of microhabitat (bulk soil, rhizosphere, and root endosphere) and irrigation on bacterial (based on weighted-UniFrac distance) and fungal (based on Bray-Curtis distance) community composition.

Factor	df	F.Model	R^2	*p*-value[†]
**Bacteria**				
Microhabitat	2	109.198	0.664	**0.001**
Irrigation	1	31.725	0.096	**0.001**
Microhabitat × Irrigation	2	11.919	0.072	**0.001**
Residuals	55		0.167	
**Fungi**				
Microhabitat	2	13.885	0.282	**0.001**
Irrigation	1	8.669	0.088	**0.001**
Microhabitat × Irrigation	2	2.067	0.042	**0.002**
Residuals	58		0.588	
**Bacteria under no-irrigation**				
Microhabitat	2	105.900	0.798	**0.001**
Cultivar	5	1.910	0.036	0.058
Microhabitat × Cultivar	10	1.109	0.042	0.382
Residuals	33		0.124	
**Fungi under no-irrigation**				
Microhabitat	2	13.243	0.335	**0.001**
Cultivar	5	2.029	0.128	**0.001**
Microhabitat × Cultivar	10	0.754	0.095	0.990
Residuals	35		0.442	

[†]*P*-value ≤ 0.05 is highlighted in bold.

Supplementary Table 2. Taxonomy information of the top 20 abundant bacterial OTUs and fungal OTUs in the root endosphere, rhizosphere, and bulk soil.

ID	Taxonomy
Bacteria	
B1	Actinobacteria|Actinobacteria|Actinomycetales|Streptomycetaceae|*Streptomyces*
B2	Proteobacteria|Alphaproteobacteria|Rhizobiales|Bradyrhizobiaceae
B3	Proteobacteria|Alphaproteobacteria|Rhizobiales|Hyphomicrobiaceae|*Rhodoplanes*
B4	Actinobacteria|Actinobacteria|Actinomycetales|Streptomycetaceae
B5	Actinobacteria|Actinobacteria|Actinomycetales|Streptomycetaceae|*Streptomyces reticuliscabiei*
B6	Proteobacteria|Alphaproteobacteria|Rhizobiales|Hyphomicrobiaceae
B7	Chloroflexi|Ellin6529
B8	Acidobacteria|Solibacteres|Solibacterales|Solibacteraceae|*Candidatus Solibacter*
B9	Actinobacteria|Actinobacteria|Actinomycetales|Micromonosporaceae
B10	TM7|TM7-3
B11	Proteobacteria|Alphaproteobacteria|Rhizobiales|Hyphomicrobiaceae|*Devosia*
B12	Chloroflexi|Ktedonobacteria|Ktedonobacterales|Ktedonobacteraceae
B13	Proteobacteria|Alphaproteobacteria|Rhodospirillales|Rhodospirillaceae
B14	Proteobacteria|Alphaproteobacteria|Rhizobiales|Bradyrhizobiaceae|*Bradyrhizobium*
B15	Proteobacteria|Alphaproteobacteria|Rhizobiales|Hyphomicrobiaceae|*Pedomicrobium*
B16	Planctomycetes|Phycisphaerae|WD2101
B17	Actinobacteria|Acidimicrobiia|Acidimicrobiales|EB1017
B18	TM7|TM7-1
B19	Proteobacteria|Alphaproteobacteria|Ellin329
B20	Proteobacteria|Alphaproteobacteria|Rhodospirillales|Rhodospirillaceae
B21	Chloroflexi|Chloroflexi|[Roseiflexales]|[Kouleothrixaceae]
B22	Actinobacteria|Actinobacteria|Actinomycetales|Propionibacteriaceae|*Microlunatus*
B23	Acidobacteria|Acidobacteriia|Acidobacteriales|Koribacteraceae
B24	Acidobacteria|Solibacteres|Solibacterales
B25	Acidobacteria|[Chloracidobacteria]|RB41|Ellin6075
B26	Chloroflexi|Ktedonobacteria|JG30-KF-AS9
B27	Proteobacteria|Alphaproteobacteria|Rhodospirillales|Rhodospirillaceae|*Reyranella massiliensis*
B28	Proteobacteria|Alphaproteobacteria|Rhizobiales|Rhizobiaceae|*Agrobacterium sullae*
B29	Actinobacteria|Actinobacteria|Actinomycetales|Mycobacteriaceae|*Mycobacterium*
B30	Actinobacteria|Actinobacteria|Actinomycetales
B31	Chloroflexi|TK10|AKYG885|Dolo_23
B32	Chloroflexi|Thermomicrobia|JG30-KF-CM45
B33	Proteobacteria|Alphaproteobacteria|Rhizobiales|Bradyrhizobiaceae|*Bradyrhizobium*
B34	Actinobacteria|Actinobacteria|Actinomycetales|Streptosporangiaceae|*Sphaerisporangium*
B35	TM7
B36	Actinobacteria|Actinobacteria|Actinomycetales|Micromonosporaceae|*Micromonospora*
B37	Actinobacteria|Actinobacteria|Actinomycetales|Pseudonocardiaceae|*Amycolatopsis*
B38	Actinobacteria|Actinobacteria|Actinomycetales|Actinosynnemataceae|*Lentzea*
B39	Acidobacteria|Acidobacteriia|Acidobacteriales|Koribacteraceae|*Candidatus Koribacter*
Fungi	

F1	Fungi
F2	Mortierellomycota|Mortierellomycetes|Mortierellales|Mortierellaceae|*Mortierella*
F3	Ascomycota
F4	Ascomycota|Sordariomycetes|Hypocreales|Sarocladiaceae|*Parasarocladium*
F5	Basidiomycota|Atractiellomycetes|Atractiellales|Hoehnelomycetaceae|*Atractiella rhizophila*
F6	Mortierellomycota|Mortierellomycetes|Mortierellales|Mortierellaceae|*Mortierella gamsii*
F7	Rozellomycota
F8	Ascomycota|Sordariomycetes|Sordariales
F9	Mortierellomycota|Mortierellomycetes|Mortierellales|Mortierellaceae|*Mortierella globulifera*
F10	Ascomycota|Dothideomycetes|Pleosporales|Cucurbitariaceae|*Pyrenochaetopsis leptospora*
F11	Ascomycota|Sordariomycetes
F12	Ascomycota|Sordariomycetes|Glomerellales|Plectosphaerellaceae|*Gibellulopsis chrysanthemi*
F13	Basidiomycota
F14	Mucoromycota|Mucoromycetes|Mucorales|Choanephoraceae|*Blakeslea trispora*
F15	Ascomycota|Sordariomycetes|Myrmecridiales|Myrmecridiaceae|*Myrmecridium schulzeri*
F16	Mortierellomycota|Mortierellomycetes|Mortierellales|Mortierellaceae|*Mortierella exigua*
F17	Ascomycota|Pezizomycotina_cls_Incertae_sedis|Pezizomycotina_ord_Incertae_sedis|Pezizomycotina_fam_Incertae_sedis|*Ciliophora*
F18	Ascomycota|Sordariomycetes|Hypocreales|Nectriaceae|*Neocosmospora falciformis*
F19	Ascomycota|Sordariomycetes|Magnaporthales|Magnaporthaceae|*Slopeiomyces cylindrosporus*
F20	Ascomycota|Dothideomycetes|Pleosporales|Didymellaceae|*Neoascochyta desmazieri*
F21	Ascomycota|Eurotiomycetes|Chaetothyriales|Herpotrichiellaceae|*Exophiala equina*
F22	Ascomycota|Sordariomycetes|Chaetosphaeriales|Chaetosphaeriaceae|*Dictyochaeta siamensis*
F23	Ascomycota|Sordariomycetes|Sordariales|Lasiosphaeriaceae|*Cercophora mirabilis*
F24	Glomeromycota|Glomeromycetes|Glomerales|Glomeraceae|*Dominikia*
F25	Chytridiomycota
F26	Ascomycota|Leotiomycetes|Helotiales|Hyaloscyphaceae|*Lachnum carneolum*
F27	Ascomycota|Sordariomycetes|Sordariales|Chaetomiaceae|*Zopfiella erostrata*
F28	Ascomycota|Sordariomycetes|Magnaporthales|Magnaporthaceae|*Pseudophialophora schizachyrii*
F29	Ascomycota|Sordariomycetes|Hypocreales|Clavicipitaceae|*Metarhizium marquandii*
F30	Ascomycota|Sordariomycetes|Hypocreales|Nectriaceae|*Mariannaea punicea*
F31	Chytridiomycota|Rhizophydiomycetes|Rhizophydiales
F32	Glomeromycota
F33	Chytridiomycota|Synchytriomycetes|Synchytriales
F34	Basidiomycota|Cystobasidiomycetes|Cystobasidiales|Cystobasidiaceae|*Occultifur*
F35	Glomeromycota|Glomeromycetes|Glomerales|Glomeraceae
F36	Chytridiomycota
F37	Basidiomycota|Agaricomycetes|Cantharellales|Ceratobasidiaceae|*Ceratobasidium*
F38	Chytridiomycota|Rhizophydiomycetes|Rhizophydiales|Rhizophydiales_fam_Incertae_sedis|*Operculomyces*
F39	Blastocladiomycota

Supplementary Table 3. Correlations between the plant quality ratings and the distance matrix of microbial communities in the root endosphere, rhizosphere, and bulk soil based on the Mantel test.

Microhabitat	Mantel Statistic R	*p*-value
**Bacteria**		
Root endosphere	0.23	**0.030**
Rhizosphere	0.07	0.248
Bulk soil	0.21	0.050
**Fungi**		
Root endosphere	0.30	**0.009**
Rhizosphere	0.27	**0.015**
Bulk soil	0.12	0.155

1.2 Supplementary Figures

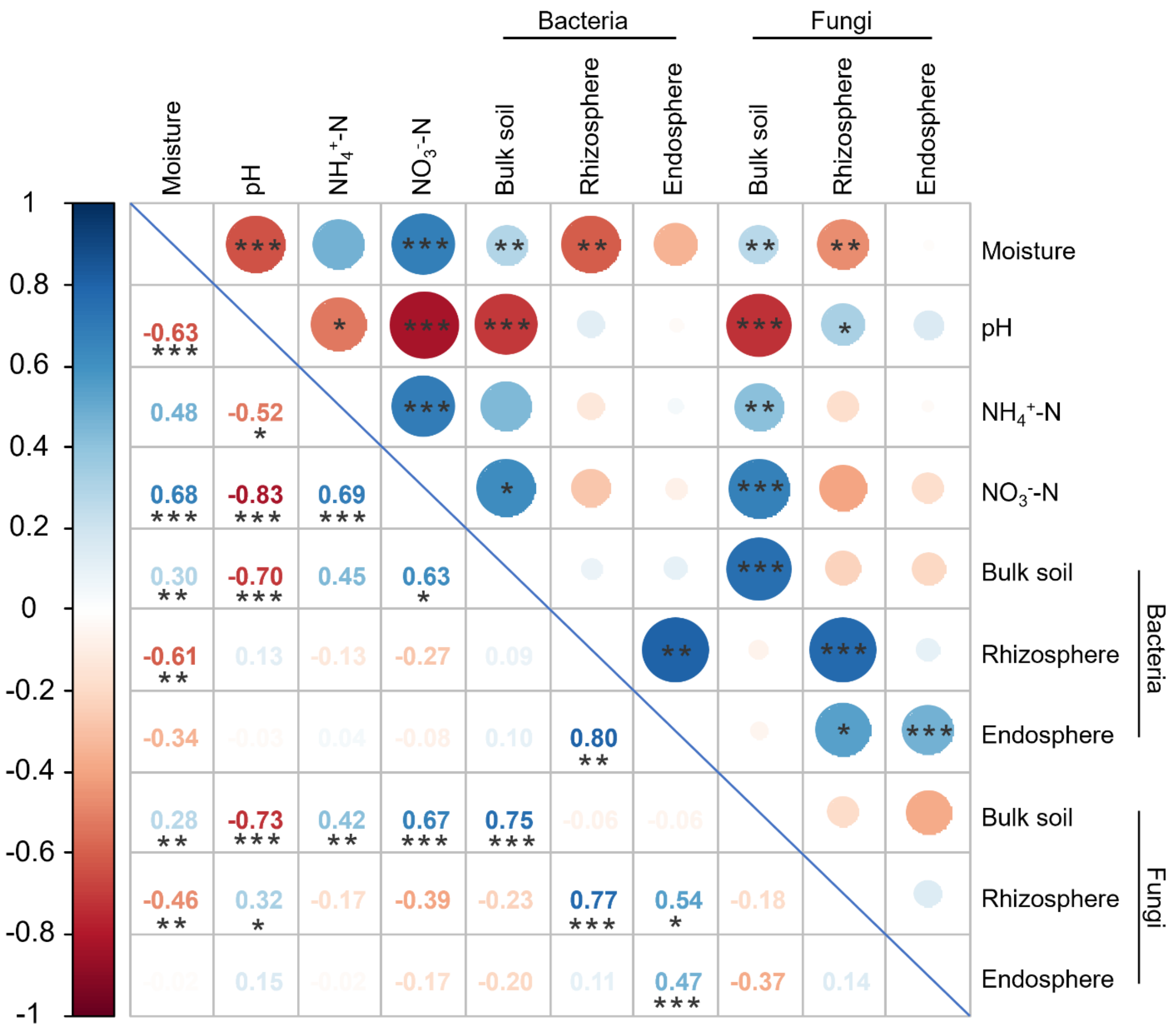

Supplementary Figure 1. The heatmap of Spearman's rank correlation coefficients between soil properties and total bacterial and fungal abundances in the bulk soil, rhizosphere, and root endosphere of tall fescue samples. The circle size and color represent the magnitude and direction of the correlation coefficient. The number represent the specific correlation coefficient. Significance is shown with asterisks. Significance levels are as follows: *, P value ≤ 0.05; **, P value ≤ 0.01; *** P value ≤ 0.001.

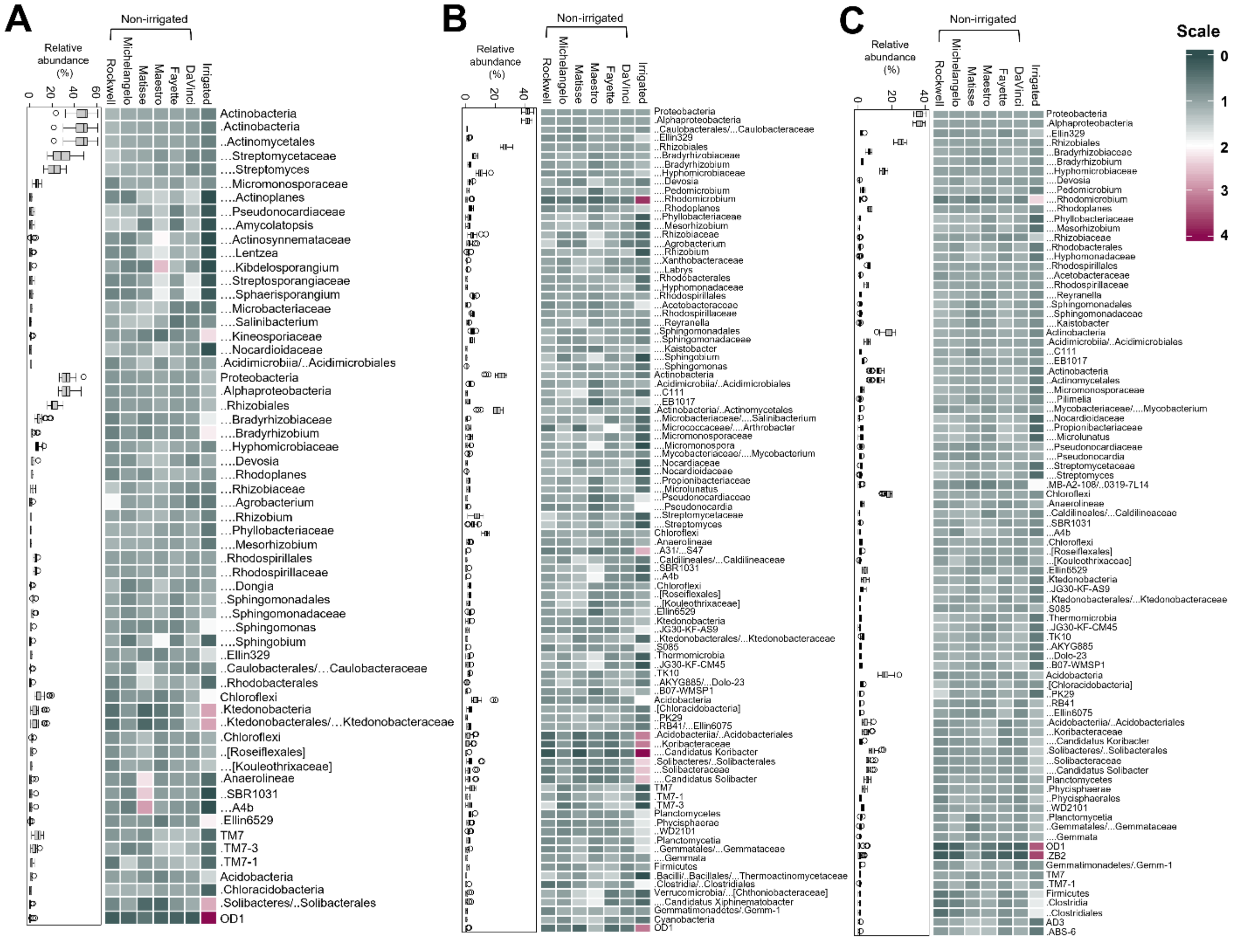

Supplementary Figure 2. Heatmaps of normalized bacterial abundances in the root endosphere (A), rhizosphere (B), and bulk soil (C). Only bacterial taxa of > 0.5% relative abundance on average across all samples were included. Normalization was made by dividing the relative abundance by the average value across all samples. Box plots represent the average values of relative abundance across all samples.

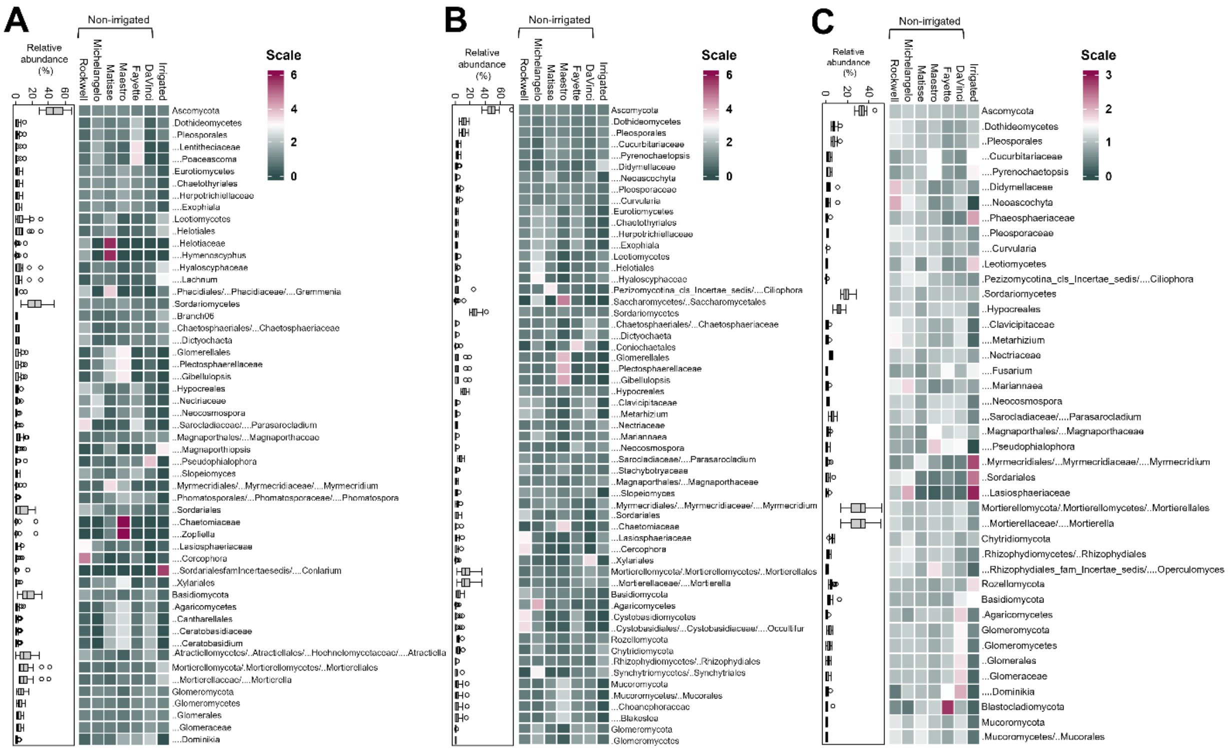

Supplementary Figure 3. Heatmaps of normalized fungal abundances in the root endosphere (A), rhizosphere (B), and bulk soil (C). Only fungal taxa of > 0.5% relative abundance on average across all samples were included. Normalization was made by dividing the relative abundance by the average value across all samples. Box plots represent the average values of relative abundance across all samples.

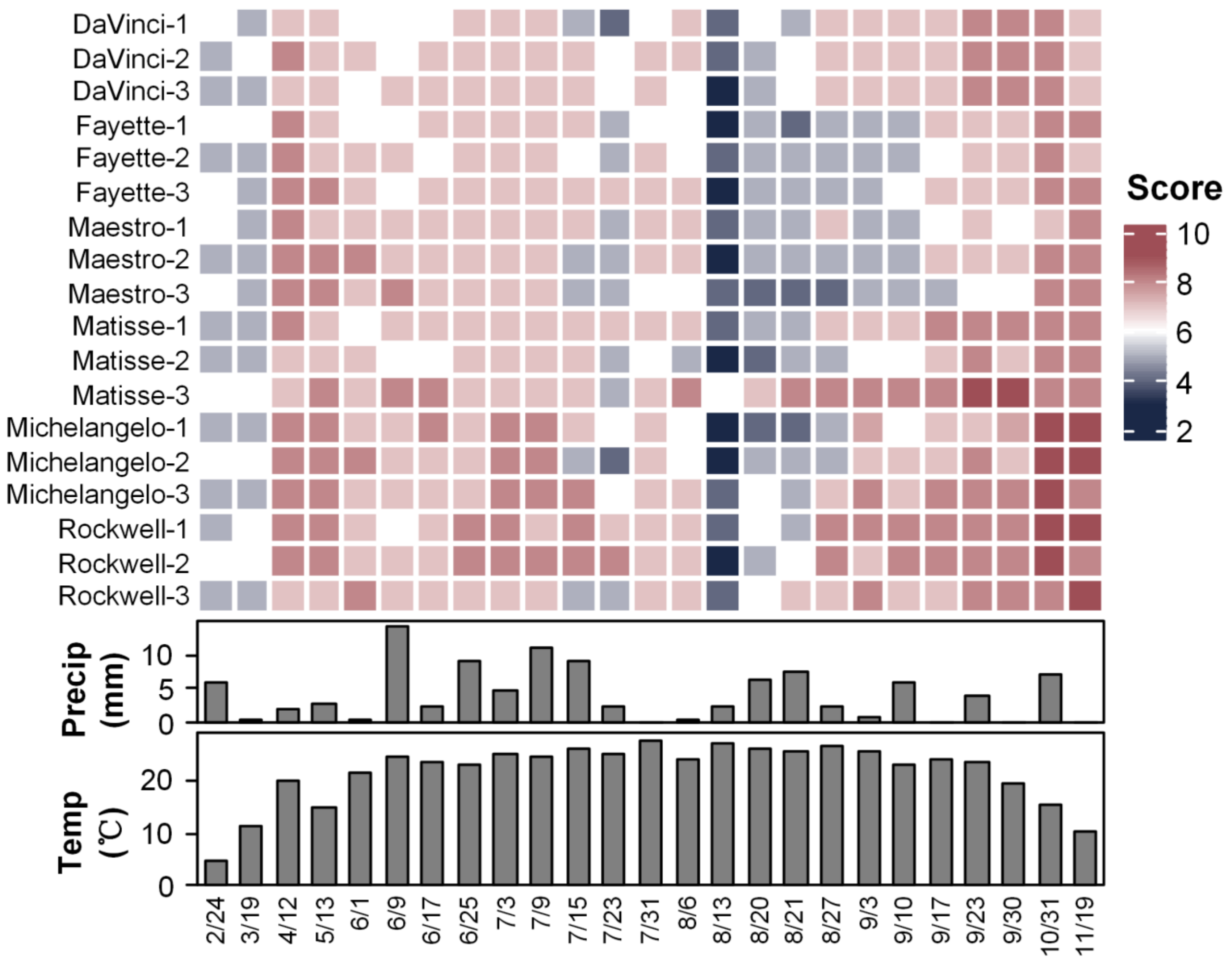

Supplementary Figure 4. Heatmap of the plant visual quality rated on a scale from 1 to 9, with 1 being a brown canopy and 9 being a dark green and uniform canopy for six tall fescue cultivars under no-irrigation during the year 2021 at irregular intervals. Bar plots represent the averaged precipitations and temperatures for seven days before each quality rating day.
